# Blockchain Integration in UAV Networks: Performance Metrics and Analysis

**DOI:** 10.3390/s24237813

**Published:** 2024-12-06

**Authors:** Md Imran Hossain, Murat Tahtali, Ugur Turhan, Kamanashis Biswas

**Affiliations:** 1 School of Systems & Computing, University of New South Wales, Canberra, ACT 2600, Australia; 2 School of Engineering & Technology, University of New South Wales, Canberra, ACT 2600, Australia; murat@unsw.edu.au; 3 School of Science, University of New South Wales, Canberra, ACT 2600, Australia; u.turhan@unsw.edu.au; 4 Peter Faber Business School, Australian Catholic University, Brisbane, QLD 4014, Australia; 5 Department of Computer Science and Engineering, Daffodil International University, Dhaka 1216, Bangladesh

**Keywords:** UAV, blockchain, throughput, latency, scalability, packet size

## Abstract

Blockchain technology has revolutionized the management of Unmanned Aerial Vehicle (UAV) networks by enhancing security, enabling decentralized control, and improving operational efficiency. This study assesses the efficiency of private blockchain architectures in UAV networks, specifically examining important performance metrics such as throughput, latency, scalability, and packet size. Furthermore, we evaluate the effectiveness of UAV networks when integrating private blockchain technologies, focusing particularly on key performance indicators such as area, altitude, and data rate. The scope of our work includes extensive simulations that employ a private blockchain to assess its impact on UAV operations. In the blockchain network, throughput decreased as the number of UAVs and transactions increased, while delay remained constant up to a certain point. In contrast, the UAV network saw improved throughput but increased delay with more UAVs and transactions. Changes in area and altitude had little impact on the blockchain network but increased delays in the UAV network. Higher data rates enhanced the UAV network by reducing latency and improving throughput, though this effect was less pronounced in the blockchain network. The aforementioned results highlight the potential and limitations of private blockchains in enhancing the durability and efficiency of UAV networks.

## 1. Introduction

Unmanned Aerial Vehicles, which are generally known as drones, have been widely integrated into various industries, including agriculture for crop monitoring, logistics for package delivery, surveillance for security purposes, and disaster management for search and rescue operations. As UAVs are versatile and efficient, they have the potential to be integrated into many aspects of our daily lives. Blockchain improves UAV networks by securing communications and enabling tamper-proof data exchange for military, airport, and border operations. It enables hangar surveillance, air traffic integration, and autonomous patrols. Blockchain enables delivery verification and optimum routes in the transportation services industry while encrypting data and automating resource allocation in agriculture. It coordinates logistics and assures responsibility in disaster assistance, while in urban air transportation, it oversees airspace and checks aircraft authorizations. However, to guarantee the flawless and secure operation of UAVs in those varieties of operations, they must have robust and efficient network management systems. This is due to the growing demand for their deployment.

Existing UAV network management solutions are generally centralized, with Ground Control Stations (GCSs) organizing and monitoring UAV activities [1]. However, the centralized strategy, which relies solely on GCSs, offers a certain level of control but also presents significant challenges:

- Security Threats: Most centralized systems are vulnerable to single points of failure, making them prime targets for cyber-attacks. Breaching the Ground Control System (GCS) could leave the whole UAV network vulnerable, potentially triggering a total breakdown [2].
- Data Integrity and Security: Ensuring data integrity and security in a centralized systems environment is challenging as the central authority is utterly responsible for all data validation and storage [3]. Unauthorized access and data tampering are potential threats associated with these types of centralized systems.
- Scalability Issues: With an increasing number of UAVs in a network, the centralized system may face challenges in managing the expanding amount of data and the connections for reliable communications among UAVs [4]. Operational efficiency may suffer as a result, and performance may also degrade.
- Operational Costs: The maintenance of a centralized infrastructure requires significant resources, such as dedicated servers, communication networks, and implementation of security measures [5]. The size and complexity of UAV operations may facilitate the acceleration of these expenses.

As a decentralized and irreversible ledger system, blockchain may provide a potential solution to these problems. The blockchain technology possesses some unique features like decentralization, immutability, and consensus procedures, which can enhance the security, reliability, and scalability of UAV networks [6]. By decentralizing control and validation among several nodes (UAVs), blockchain technology can effectively mitigate the single point of failure, therefore minimizing the vulnerability to cyber-attacks and ensuring the integrity of data.

The idea of integrating blockchain with UAV networks has gained considerable attention in recent years. Blockchain is classified into three major types: public, private, and consortium. Public blockchains including Bitcoin and Ethereum are decentralized, permissionless networks that allow anybody to join and contribute. Consortiums, conversely, are somewhat decentralized and regulated by one or more organizations, often used in commercial collaborations. In contrast, private blockchains are permissioned networks where only authorized participants can join and take part in transaction validation. This blockchain type is particularly important for sensitive applications such as Mobile Ad hoc Networks (MANETs) and UAV networks due to its dynamic network partitioning, privacy, control, and security properties. UAV networks, frequently transmitting sensitive information and functioning in dynamic environments, demand robust security to prevent illegal access and maintain communication integrity [7]. However, the majority of research has focused on public blockchains, which, despite their robustness, may not be suitable for UAV networks due to their inherent limitations in terms of transaction speed and scalability. Private blockchains, on the other hand, offer a more controlled and scalable environment, making them an attractive option for UAV applications.

Implementing blockchain in UAV networks faces several limitations and challenges that need to be addressed for seamless integration and operation. Blockchain transactions may introduce latency due to consensus mechanisms, impacting real-time UAV operations such as collision avoidance or dynamic route adjustments. High-frequency data from large UAV networks can overwhelm blockchain networks, raising scalability concerns. Ensuring secure key management for UAVs in the blockchain network is critical but can be a challenge. Additionally, blockchain’s inherent design prioritizes immutability and security over speed, which may hinder its ability to handle real-time UAV data and decision-making [8]. Energy-intensive consensus mechanisms such as PoW are unsuitable for UAVs, necessitating the development of lightweight alternatives.

This paper aims to evaluate the performance of private blockchain implementations in UAV networks, focusing on key performance indicators such as latency, throughput, scalability, and security. We employ a private blockchain platform to conduct extensive simulations in a controlled UAV network environment. Our study aims to provide a comprehensive understanding of the potential benefits and limitations of private blockchain technology in enhancing the robustness and efficiency of UAV network operations.

The rest of the paper is organized as follows: Section 2 offers an overview of the related works. Section 3 presents the system overview with architecture and performance metrics. In Section 4, the proposed scheme is exhibited. Section 5 depicts the results and discussion on the performance evaluation. Finally, Section 6 concludes the paper.

## 2. Related Works

This literature review explores various blockchain-based approaches proposed by researchers to address the challenges of secure communication, data integrity, and scalability in UAV networks. Additionally, these works highlight the importance of performance metrics, such as throughput, latency, energy consumption, and scalability, in evaluating the effectiveness of blockchain systems in dynamic UAV environments. The work aims to provide an overview of the state-of-the-art solutions and their potential to transform UAV communication systems by leveraging the decentralized and secure nature of blockchain technology. Wang et al. [9] propose a decentralized system for UAV networks using blockchain technology. They incorporate committee elections and clustering optimizations into their system to reduce the complexity of communication and enhance the scalability of blockchain. In order to secure efficient energy consumption and scalability of UAV networks, the framework undergoes security analysis and performance evaluation. Furthermore, they implement sharding and clustering techniques to enhance communication efficiency and minimize energy usage. Their simulation findings demonstrate that the approach exhibits reduced energy consumption and achieves a linear increase in throughput as the network size grows.

Ghribi et al. [10] develop a novel approach to improve the security of communications in UAV networks by employing blockchain technology. The authors present a blockchain-based communication system that ensures security, decentralization, and reliable data transfer among UAVs. Their proposal suggests a novel consensus algorithm named Proof-of-Communication, which combines blockchain technology with sophisticated cryptographic techniques, like Elliptic Curve Diffie–Hellman (ECDH) and one-time pad (OTP) encryption. The analysis highlights that while blockchain offers several benefits, its implementation in UAV networks necessitates careful consideration of network scalability, latency, and computation costs.

Gai et al. [11] present a blockchain-enabled solution to facilitate multi-party authentication in order to enhance the security of group communications within UAV networks. The authors argue that their attribute-based system has the ability to improve UAV authentication, therefore ensuring that only authorized devices are able to participate in communications. The blockchain system can record communication activities, therefore ensuring traceability and secure data transmission, while simultaneously protecting against threats like spoofing and denial of service (DoS). The experiments demonstrate the system’s performance, with low execution time and gas costs during the validation of group communications in UAV networks.

Bera et al. [12] demonstrate a blockchain-based secure data delivery and collection scheme (BSD2C-IoD) for Internet of Drones (IoD) environments. The system boosts security by using blockchain to manage communications among drones, ground stations, and control rooms, therefore assuring the integrity of data and shielding against threats such as man in the middle, replay, and impersonation. Simulation and security analysis are used to validate the scheme’s effectiveness, showing that it has less communication and computation overhead than alternative approaches.

Aloquaily et al. [13] exhibit a guideline for blockchain-assisted 5G-UAV networks, focusing on enhancing smart city services through decentralized, secure, and efficient communication. By integrating blockchain with UAVs, the system improves data security, trust, and privacy while ensuring reliable data delivery and resource management. UAVs act as mobile access points, connecting with edge and cloud computing resources to support various smart city applications. The study also compares the data delivery success rate and number of messages exchanged using the proposed solution and other state-of-the-art UAV-supported data delivery techniques.

Hossain et al. [14] analyze the performance of private blockchain implementation in Mobile Ad hoc Networks (MANETs), aiming at some key metrics like throughput, latency, and scalability. The paper investigates the performance of blockchains in static and dynamic MANET environments employing the Practical Byzantine Fault Tolerance (PBFT) consensus algorithm. The findings demonstrate that static networks exhibit superior throughput compared to dynamic networks while retaining scalability up to 100 nodes with tolerable latency. Furthermore, the research emphasizes the influence of data rate, area size, packet size, and hashing on the performance of blockchains, proposing enhancements in hashing speed to achieve greater overall efficiency.

Ahanger et al. [15] propose a distributed blockchain-based platform for unmanned aerial vehicles (UAVs) aimed at enhancing security and coordination in IoT environments. The platform addresses the challenges of data integrity, privacy, and operational autonomy by using a lightweight blockchain architecture. It introduces a reputation-based consensus mechanism to ensure the trustworthiness of UAV communications, mitigating risks such as Sybil attacks, DoS, and GPS spoofing. Performance evaluations demonstrate the system’s statistical effectiveness in the form of temporal delay, packet flow efficacy, precision, specificity, sensitivity, and security efficiency.

Feng et al. [16] suggest a wireless UAV–blockchain system where each UAV acts as a blockchain node, performing transactions, interactions, and consensus mechanisms via wireless ad hoc channels. The study analyses the performance of the system, focusing on throughput, block generation rates, and the effects of network parameters such as UAV density and transaction arrival rates. Results show that while higher transaction rates can introduce blockchain forks and reduce system efficiency, the UAV–blockchain system maintains effective performance in handling data exchanges and securing communications. The paper highlights how UAV density and network throughput impact blockchain consistency and performance in such distributed systems.

García-Magariño et al. [17] recommend an agent-based approach inspired by blockchain principles to enhance security in networks of unmanned aerial vehicles (UAVs) used for surveillance. By utilizing secure asymmetric encryption and a peer-to-peer information-sharing model, the system effectively detects and neutralizes misinformation.

Allouch et al. [18] illustrate UTM-Chain, a blockchain-based security solution for unmanned traffic management (UTM) in the Internet of Drones (IoD). UTM-Chain addresses key security challenges such as data integrity, privacy, and availability while mitigating risks like GPS spoofing and communication jamming. The proposed solution is efficient and scalable, providing a secure and tamper-resistant platform for managing drone traffic in low-altitude airspace. The performance of the proposed system is evaluated by computing the transaction delay and resource utilization using cAdvisor.

Chen et al. [19] provide an experimental study on the performance of private blockchain in IoT applications, specifically focusing on Ethereum. The study evaluates key performance metrics such as latency and resource usage (CPU, memory, disk, and network throughput) in both indoor IoT environments and cloud-based deployments. Results show that latency increases with the number of network hops and varies between transaction-oriented and block-oriented processes. Additionally, resource consumption is manageable for non-mining IoT devices, while mining processes require significant computational power.

Sun et al. [20] explore a blockchain-enabled wireless IoT system, focusing on performance analysis and optimal communication node deployment. They establish a model using the Poisson Point Process to evaluate key metrics like signal-to-interference-plus-noise ratio (SINR), transaction success rates, and throughput. The study also presents an algorithm to optimize node deployment to maximize transaction throughput. Security analysis is included, addressing typical attacks such as eclipse, random link, and random node attacks, demonstrating the system’s resilience through physical layer security measures and blockchain protocols.

Alrubei et al. [21] present an experimental study on the latency and performance of real-world wireless IoT blockchain applications, specifically using Ethereum’s Proof of Authority (PoA) consensus protocol. The study focuses on measuring transaction arrival times, end-to-end system latency, and the energy consumption of IoT devices in both Wi-Fi and 3G cellular network environments. Results show that shorter block periods can lead to higher delays, especially in cellular networks, affecting synchronization and overall system stability.

Lee et al. [22] analyze the performance of blockchain systems with wireless mobile miners (MMs), focusing on the impact of transmission latency and energy consumption in a mobile environment. The study proposes a framework in which MMs handle the computational tasks while communication nodes (CNs) store the blockchain ledger. The research highlights that increasing the number of MMs can significantly reduce energy consumption and improve efficiency. However, high transmission latency between MMs and CNs increases the likelihood of forking events, which negatively affects blockchain performance.

Ferrag et al. [23] provide a tutorial on performance evaluation techniques for blockchain-based security and privacy systems in the Internet of Things (IoT). the work reviews existing blockchain-based solutions across various IoT applications, such as smart cities, healthcare, and energy, and compares consensus algorithms in terms of latency, throughput, scalability, and security. The study also presents cryptographic libraries and blockchain testbeds used for IoT system performance evaluation and outlines key challenges in implementing blockchain solutions for IoT.

The blockchain-envisioned UAV network addresses environmental variances such as weather and signal interference. Advanced wireless technologies like 4G, 5G, and Wi-Fi with error correction and multipath transmission can ensure reliable data exchange, while dynamic channel allocation and adaptive modulation optimize performance under varying conditions [24]. The PBFT consensus mechanism enhances resilience by tolerating faulty or delayed nodes, and the system allows delayed synchronization for UAVs temporarily disconnected due to environmental disruptions. UAVs employ real-time monitoring and adjust transmission power or routes to maintain connectivity, while redundant ledger copies and delayed block propagation ensure blockchain consistency. Onboard sensors monitor environmental factors like wind and visibility, enabling UAVs to adapt flight paths dynamically. Additionally, local computation via edge computing can reduce the reliance on continuous communication, while robust security protocols like data hashing preserve data integrity against interference [25]. Collectively, these measures may ensure the UAV blockchain network remains secure, consistent, and operational despite environmental challenges.

## 3. System Overview

**Figure 1:**
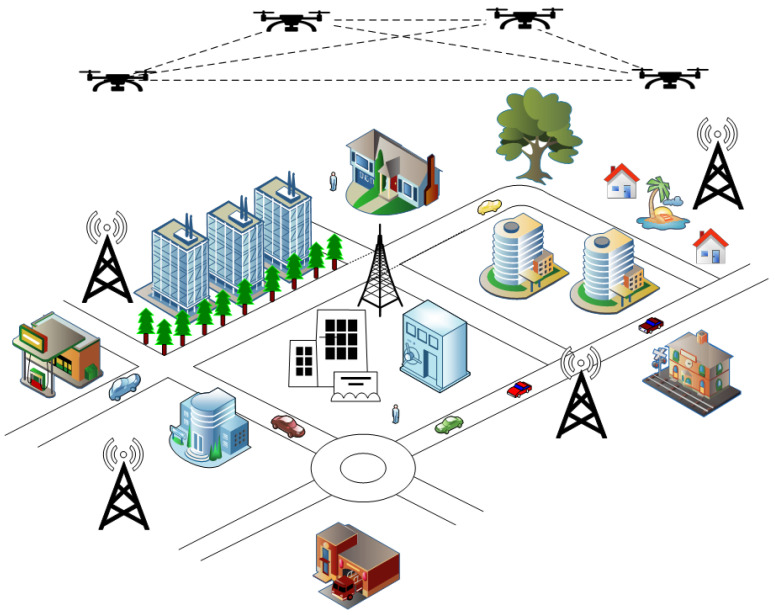
Blockchain-envisioned UAV network.

Integrating blockchain into a UAV network offers some opportunity for a decentralized and secure network where UAVs can act as blockchain nodes and can communicate between them, exchange data and coordinate missions without the help of a centralized authority. Figure 1 shows a generic architecture of a blockchain-envisioned UAV network. The proposed system uses a layered architecture to manage different components, including hardware, communication, consensus, and blockchain storage, ensuring efficiency and security.

### 3.1. System Components

This system intends to measure the performance of blockchain when integrated into UAV networks. Integration of blockchain into UAV networks may provide data integrity and decentralized control, which are critical in UAV networks. However, this integration may lead to performance issues in both blockchain and UAV networks, such as increased computational overhead, latency, and resource consumption. The system is designed to analyze and evaluate these factors, providing insights into the feasibility and effectiveness of blockchain in UAV networks.

- UAVs (Drones): Each UAV is equipped with different sensors, processing units, and communication modules capable of running blockchain inside it. These UAVs establish a decentralized network in which every node is actively included in the blockchain. UAVs can be designed as either full nodes or lightweight nodes. If the UAVs are configured as full nodes, then they can maintain a complete copy of the blockchain ledger and can participate fully in the consensus process. Alternatively, the UAV nodes can be configured as lightweight nodes, and then the UAV nodes might only store essential parts of the blockchain and rely on nearby full nodes for transaction verification, reducing computational complexity and storage burden. In our system, we used lightweight UAV nodes to measure the performance of both blockchain and UAV networks.
- Blockchain Network: The blockchain network operates across the UAVs where each UAV can be configured as a node within the blockchain. This implies that every UAV carries out its main tasks such as surveillance, delivery, or mapping while simultaneously engaging in the blockchain network by preserving a copy of the blockchain ledger and contributing to the transaction validation process or, more precisely, the consensus mechanism. To ensure data consistency and reliability in this decentralized network, we employed the PBFT mechanism as a consensus method.
- Ground Control Station (GCS): The Ground Control System (GCS) typically manages and regulates the UAV network. When blockchain is implemented in UAV networks, it engages with the blockchain to authenticate UAVs, ensure data integrity, issue commands, and retrieve stored data. Considering the potential for a single point of failure, we did not consider GCS in our operational model. While GCS exists within our system, it does not actively participate in the core operations.

### 3.2. Layered Architecture

**Figure 2:**
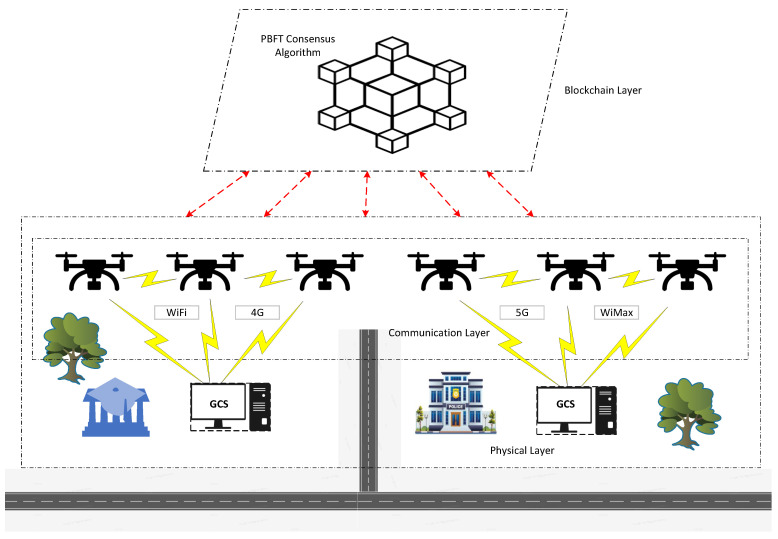
Layered architecture of the proposed model.

When blockchain is integrated into a UAV network, the system is structured into several key layers that enable decentralized operation and secure data management as shown in Figure 2. Each layer has a distinct role in ensuring the proper functioning and efficiency of the overall network.

- Physical Layer: The hardware layer forms the foundational level of the UAV network, comprising essential components such as sensors, cameras, and processing units. Each UAV is equipped with a range of sensors and cameras to collect real-time data, such as environmental conditions or visual information for tasks like surveillance, mapping, and monitoring [26]. The onboard processing units handle the computational tasks, including executing blockchain operations such as transaction validation and data encryption. This layer is crucial as it gathers raw data, which is then processed, stored, or transmitted to higher layers for further action, ensuring that the UAVs can effectively monitor and interact with their surroundings.
- Communication Layer: The communication layer is responsible for ensuring seamless data transmission between UAVs. It consists of wireless communication modules and utilizes advanced technologies like WiFi, 4G and 5G to facilitate long-range, high-speed communication. UAVs exchange status updates, sensor data, and transactions over these networks. This layer ensures the reliability and efficiency of data exchange between nodes, enabling UAVs to coordinate tasks and share critical information in real-time, even across large distances [27]. The communication layer is the backbone of a decentralized network like blockchain, allowing UAVs to operate collaboratively without the need for a centralized controller.
- Blockchain Layer: The blockchain layer is responsible for securely storing validated transactions in the form of a blockchain ledger. This layer ensures that data are immutable and securely stored across the network. In a decentralized system like a UAV network, multiple nodes (UAVs) must agree on the state of the blockchain to maintain consistency and security which is known as consensus protocol. The PBFT consensus algorithm is one of the most commonly used protocols in wireless blockchain environment [7]. We employed PBFT as the consensus algorithm for the blockchain layer in our system. PBFT enables the network to reach consensus even when some nodes are faulty or compromised. This layer plays a key role in maintaining trust and reliability within the decentralized UAV network.

## 4. Proposed Scheme

### 4.1. Initialization

During the initialization phase, the UAV network and blockchain infrastructure are established. This stage includes deploying UAVs, configuring communication channels, and initializing the blockchain system to ensure that the network is ready for operation. UAVs are deployed across a designated geographic region to create an ad hoc network, with each UAV functioning as a node within the blockchain system. The deployment follows a Poisson Point Process (PPP) [28], a stochastic model that represents the random placement of UAV nodes across the area.

(1) $$f_{R_{u}}\left(r_{u}\right)=\frac{r_{u}e^{-r_{u}^{2}/\left(2\sigma_{u}^{2}\right)}}{\sigma_{u}^{2}\left(1-e^{-r_{M}^{2}/\left(2\sigma_{u}^{2}\right)}\right)}\cdot 1\left(r_{u}<r_{M}\right)$$

This equation models the probability density function ($f_{R_{u}}\left(r_{u}\right)$) of distances $r_{u}$ between a UAV transmitter and its corresponding receiver, considering factors like the maximum transmission distance $r_{M}$ and the Rayleigh scale parameter $\sigma_{u}$, which is related to the average distance between nodes. Exponential term ($e^{-r_{u}^{2}/\left(2\sigma_{u}^{2}\right)}$) indicates the probability decay with distance $r_{u}$, representing the Rayleigh distribution.

Next, the communication channels are initialized to establish reliable links between the UAVs. This ensures efficient transmission of data including transactions and blocks across the network.

(2) $$P_{r}=P_{t}\cdot g_{t}\cdot g_{c}\cdot g_{h}$$

Here, the received signal strength $P_{r}$ depends on the transmit power $P_{t}$, the antenna gains $g_{t}$ and $g_{c}$, and the channel power gains $g_{h}$.

After initializing the UAV network, the blockchain is set up. The process begins with generating the genesis block, which acts as the foundation for all subsequent blocks in the blockchain infrastructure.

$$GenesisBlockB_{0}=\mathrm{blockchain}.\mathrm{generate}\_\mathrm{genesis}\_\mathrm{block}\left(\right)$$

Once both the UAV network and blockchain are initialized, the UAVs are registered with the blockchain network. The UAVs then generate data, such as sensor readings, flight logs, or other operational information, which are recorded as transactions on the blockchain. However, to reduce storage space usage, only data hash can be stored on-chain, whereas the original data are sent to the GCS for storage in off-chain systems such as the Interplanetary File System (IPFS). The *Poisson Proces* for transactions can be defined as follows:

$$\lambda_{t}=\mathrm{Average}\;\mathrm{transaction}\;\mathrm{generation}\;\mathrm{rate}\;\mathrm{per}\;\mathrm{UAV}$$

The rate $\lambda_{t}$ represents how frequently each UAV generates transactions. This process follows a Poisson distribution, which is commonly used to model the occurrence of events over time.

To create blocks from the generated transactions, a set number of transactions are grouped into a block. Once the block is formed, it is added to the blockchain after the network reaches consensus. The block arrival rate is

(3) $$\lambda_{b}=\lambda_{t}\times S$$

The block arrival rate $\lambda_{b}$ is determined by the transaction generation rate $\lambda_{t}$ and the number of transactions per block *S*. This rate influences how quickly the blockchain grows and how frequently blocks are mined.

Consensus ensures that all UAVs in the network agree on the addition of new blocks to the blockchain, preventing the emergence of conflicting versions and maintaining a unified ledger across the network. Consensus probability is

(4) $$\Theta =\mathrm{Pr}\left(\tau_{1}<\frac{1}{\lambda_{b}}\right)\lambda_{b}+\sum_{i=2}^{\infty }\left\{\prod_{k=1}^{i-1}\mathrm{Pr}\left(\tau_{k}>\frac{k}{\lambda_{b}}\right)\mathrm{Pr}\left(\tau_{i}\leq \frac{i}{\lambda_{b}}\right)\frac{\lambda_{b}}{i}\right\}$$

Here, Θ represents the probability that a block is successfully added to the main blockchain. This probability depends on factors like the block transmission time $\tau_{i}$ and the block arrival rate $\lambda_{b}$. Achieving consensus is critical for maintaining the integrity and security of the blockchain.

Algorithm 1 initializes a Wi-Fi network infrastructure comprising Access Point (AP) and Station (STA) nodes, sets up Wi-Fi channel and PHY properties, and configures tracing and monitoring mechanisms. It installs the Internet stack on all nodes, assigns IP addresses, and sets up UDP echo server and client applications. The algorithm enables network animation and tracing and starts a flow monitor to capture packets. Finally, it collects throughput and delay statistics from the flow monitor and prints the results. The network initialization involves configuring AP and STA nodes using NodeContainer, with YANS Wi-Fi channel and YansWifiPhyHelper to simulate realistic wireless conditions. The MAC layer is set up via WifiMacHelper, using a constant data rate with ns3::ConstantRateWifiManager. AP and STA nodes are configured with a common SSID and equipped with the Internet stack, with IPv4 addresses assigned using a predefined base and subnet mask. Node mobility is modelled using a Gauss–Markov mobility model, influencing node positions and network dynamics. UDP echo servers and clients simulate application-level interactions, while PCAP tracing and AnimationInterface provide detailed packet-level visibility. A flow monitor captures key performance metrics such as throughput and delay, facilitating a comprehensive analysis of network behavior. The simulation framework is used to execute the setup, ensuring a rigorous evaluation of PHY/MAC settings and mobility impacts on network performance.

### 4.2. Communication

The Communication Phase involves the transmission of transactions and blocks across the UAV network. To transmit blocks between UAVs over the network, ensuring that all nodes have a consistent view of the blockchain is essential. Transmission time is calculated as

(5) $$\tau =\frac{C}{r_{u}}$$

The time τ required to transmit a block is directly proportional to the block size *C* and inversely proportional to the data rate $r_{u}$ between UAVs. Efficient transmission is crucial for maintaining high throughput and low latency in the network.

To determine the likelihood that a block will be successfully generated and added to the main chain without causing forks or conflicts, the following probability equation is used:

(6) $$\Theta =\mathrm{Pr}\left(r_{u}\geq C\lambda_{b}\right)\lambda_{b}+\sum_{i=2}^{\infty }\left\{\prod_{k=1}^{i-1}\mathrm{Pr}\left(r_{u}<\frac{C\lambda_{b}}{k}\right)\mathrm{Pr}\left(r_{u}\geq \frac{C\lambda_{b}}{i}\right)\frac{\lambda_{b}}{i}\right\}$$

This equation calculates the probability Θ that a block will be added to the blockchain successfully. It accounts for factors like data rate $r_{u}$, block size *C*, and block arrival rate $\lambda_{b}$. This metric is vital for ensuring that the blockchain remains consistent and secure across all UAVs. The provided Algorithm 1 illustrates the initialization process of the network.
**Algorithm 1** Network Initialization 1:**Function** InitializeNetwork(nod, datarate, packetSize) 2:     # Set up nodes, Wi-Fi channel, Wi-Fi MAC, mobility model, and configure tracing and monitoring 3:     InitializeApNodes() 4:     initializeStaNodes() 5:     # Set up Wi-Fi channel and PHY properties 6:     channel = YansWifiChannel() 7:     phy_helper = YansWifiPhyHelper() 8:     SetupSsid() 9:     SetupMac()10:   SetupDataRate(’fdmRate’ in Mbps)11:   SetRemoteStationManager(“ns3::ConstantRateWifiManager”)12:   # Configure STAs and AP13:   mac.SetType(“ns3::ApWifiMac”, “Ssid”)14:   ConfigureApDevice()15:   ConfigureStaDevice()16:   # Set up mobility model and calculate mobility17:   SetMobilityModel(“ns3::GaussMarkovMobilityModel”, “Bounds”)18:   mobility.Install(all_nodes)19:   Calculate(mobility)20:   Calculate(Area)21:   # Install Internet stack on all nodes and assign IP addresses22:   stack.Install(all_nodes)23:   all_nodes.SetBase(Ipv4Address(“10.1.1.0”), Ipv4Mask(“255.255.255.0”))24:   # Set up UDP echo server on AP25:   serverApps = echoServer.Install(ap_node.Get(0))26:   # Set up UDP echo client on STAs27:   clientApps = echoClient.Install(sta_nodes)28:   # Enable network animation29:   anim = AnimationInterface()30:   # Set up network tracing31:   phy_helper.EnablePcap()32:   # Set up flow monitor to capture packets and begin simulation33:   StartFlowMonitor()34:   # Collect throughput and delay stats35:**for** each flow_id, flow_stats in stats **do**36:   Print flowStatistics()37:**end for**38:End Simulation

The blockchain processing Algorithm 2 executes transaction handling, consensus achievement, and block mining. It is initialized by loading the blockchain state and generating a new unmined block linked to the latest block. Consensus parameters are determined using Byzantine fault tolerance, setting the required number of nodes (2*f* + 1) based on the potential faulty nodes (*f* = (nodes − 1) / 3). The block proposal is broadcast, and prepared confirmations are collected. If the count of prepared nodes meets the required threshold, the consensus is validated. Upon achieving consensus, and provided the node is non-faulty, the block is mined and disseminated across all nodes. If consensus fails or nodes are faulty, the block is not mined, ensuring integrity and consistency in the blockchain network.
**Algorithm 2** Processing Blockchain and Achieving Consensus 1:**Function** ProcessBlockchain() 2:blockchain = LoadBlockchain() 3:last_block = GetLastBlock(blockchain) 4:unmined_block = CreateUnminedBlock(last_block) 5:# Determine consensus requirements based on the number of nodes 6:faultyNodes = (nodes − 1) / 3 7:requiredNodes = (2 × faultyNodes) + 1 8:# Broadcast block proposal and collect prepared responses 9:broadcastResult = BroadcastProposal(unmined_block)10:preparedNodes = CollectPreparedConfirmationMessages(broadcastResult, nodes)11:# Consensus logic to ensure all prepared nodes agree on the block12:**if** CountPreparedNodes(preparedNodes) ≥ requiredNodes **then**13:   consensusResult = AchieveConsensus(preparedNodes, unmined_block)14:   **if** consensusResult == True **then**15:     **if** currentNode **not in** faultyNodes **then**16:        MineBlock(blockchain, unmined_block)17:        UpdateBlockchainForAllNodes()18:        miningResult = “Block mined successfully”19:     **else**20:        miningResult = “Current node is faulty, cannot mine”.21:     **end if**22:   **else**23:     miningResult = “Consensus not reached, cannot mine”.24:   **end if**25:**else**26:   miningResult = “Not enough ready nodes, cannot mine”.27:**end if**

### 4.3. Performance Measurement

To measure the overall efficiency of the blockchain network in terms of how many transactions it can process per second, throughput (TPS) is calculated:

(7) TPS=Θ×S

*Transactions Per Second (TPS)* is a key performance indicator for the blockchain. It is calculated as the product of the block generation probability Θ and the number of transactions per block *S*. Higher *TPS* indicates a more efficient and scalable blockchain system.

To measure latency, the total time required for a transaction to be confirmed within a blockchain network is required. Latency *(L)* is calculated as follows:

$$L=\frac{B_{s}}{R}+\frac{N_{t}\cdot T_{s}}{R}+D_{p}$$

The latency *L* represents the time delay between the submission of a transaction and its confirmation on the blockchain. Here, $B_{s}$ is the block size, *R* denotes the transmission rate or bandwidth, and $N_{t}$ is the number of transactions in a block. $T_{s}$ is the average size of each transaction and $D_{p}$ is for the propagation delay across the network.

The performance measurement Algorithm 3 calculates throughput and delay for both network flows and blockchain transactions. Network metrics are obtained by evaluating each flow’s statistics and aggregating total and average throughput and delay. Similarly, blockchain metrics are calculated by analyzing each transaction’s performance, summarizing total and average throughput and delay. This method provides a precise assessment of network and blockchain efficiency, capturing key performance indicators across both domains within the simulation.
**Algorithm 3** Throughput and Delay measurement for UAV Network and Blockchain 1:**Function** MeasurePerformance(flows, simulationTime, blockchainTransactions) 2:# Collect network throughput and delay 3:**for** each flow in flows **do** 4:   throughput, delay = CalculateFlowStats(flow) 5:   throughputList.append(throughput) 6:   delayList.append(delay) 7:**end for** 8:# Collect blockchain throughput and delay 9:**for** each transaction in blockchainTransactions **do**10:   blockchainThroughput, blockchainDelay = CalculateBlockchainStats(transaction)11:   blockchainThroughputList.append(blockchainThroughput)12:   blockchainDelayList.append(blockchainDelay)13:**end for**14:# Calculate Network Performance Metrics15:totalThroughput = sum(throughputList)16:**if** throughputList Not Empty **then**17:   averageThroughput = totalThroughput / len(throughputList)18:**else**19:   averageThroughput = 020:**end if**21:totalDelay = sum(delayList)22:**if** delayList Not Empty **then**23:   averageDelay = totalDelay / len(delayList)24:**else**25:   averageDelay = 026:**end if**27:# Calculate Blockchain Performance Metrics28:blockchainTotalThroughput = sum(blockchainThroughputList)29:**if** blockchainThroughputList Not Empty **then**30:   blockchainAverageThroughput = blockchainTotalThroughput / len(blockchainThroughputList)31:**else**32:   blockchainAverageThroughput = 033:**end if**34:blockchainTotalDelay = sum(blockchainDelayList)35:**if** blockchainDelayList Not Empty **then**36:   blockchainAverageDelay = blockchainTotalDelay / len(blockchainDelayList)37:**else**38:   blockchainAverageDelay = 039:**end if**

## 5. Results and Discussion

In this section, we evaluate the performance of our proposed blockchain-based UAV network using a series of experiments. The focus of the analysis is on key performance metrics such as throughput, latency, area, height, and data rate under various conditions.

### 5.1. Performance Metrics

The system analyses the essential performance indicators such as throughput and latency for both blockchain and UAV networks under various conditions including changes in the number of nodes, transactions, operating area, and height of UAVs. A higher throughput indicates better performance in managing substantial amounts of data. Whereas latency is crucial for time-sensitive UAV operations. By measuring throughput and latency, the system assesses the network’s scalability, determining satisfactory performance levels which is very important for implementing blockchain in UAV networks. Furthermore, it provides a prospective viewpoint for researchers on the incorporation of blockchain technology into UAV networks, including the selection of appropriate UAV networks for integration.

### 5.2. Experiment Setup

The proposed architecture was evaluated using NS-3.36.1 [29], a discrete-event network simulator. The simulations were run on a machine equipped with an 11th Gen Intel(R) Core(TM) i5-1145G7 processor, operating at a frequency of 2.60 GHz and 12 GB RAM. The UAVs were modelled as nodes within the blockchain network, with varying numbers of UAVs and transactions to a 100 m × 100 m × 100 m region. In the simulation, we considered a range of UAVs from 5 to 100 and transaction counts of 10, 20, 50, 100, 150, and 200. The Gauss–Markov Mobility Model was integrated with UAV mean velocities ranging from 800 to 1200 m/s. The duration of each simulation experiment was 100 s, and each experiment was run 10 times. Thus, results were computed as an average of 10 runs. Table 1 presents the simulation parameters used in the experiments.

### 5.3. Throughput and Latency Measurement

We evaluated the throughput and latency of both the blockchain network and the UAV network under varying conditions. The performance of both networks was assessed by changing the number of UAVs and the number of transactions.

**Figure 3:**
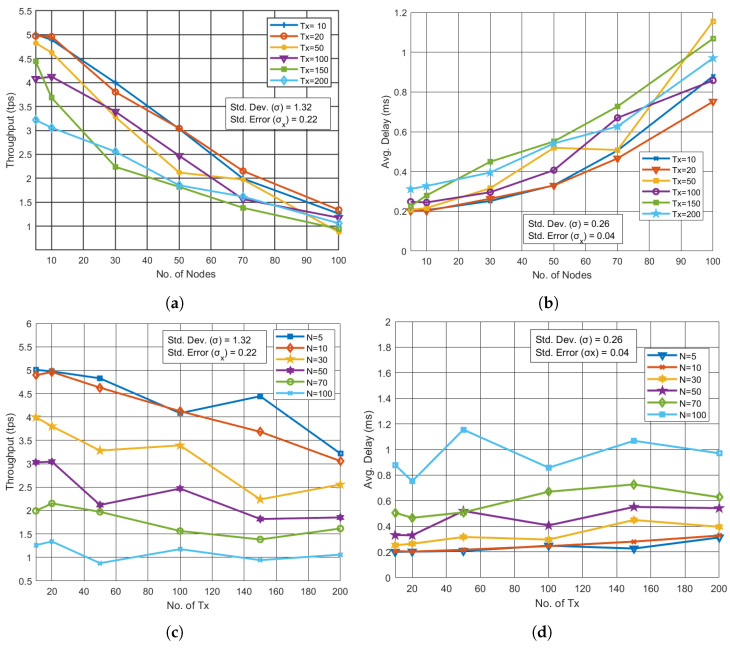
Performance analysis for varying nodes and transactions in BC. (**a**) Throughput vs. No. of UAVs with varying Tx. (**b**) Avg. Delay vs. No. of UAVs with varying Tx. (**c**) Throughput vs. No. of Tx with varying UAVs. (**d**) Avg. Delay vs. Tx with varying UAVs.

For the blockchain network, throughput decreased as the number of UAVs and transactions increased. We varied the number of UAVs from 5 to 100, as shown in Figure 3a,b. With an increase in UAVs from 5 to 100, the throughput dropped from 5 TPS to 0.8 TPS for fixed transaction counts of 10, 20, 50, 100, 150, and 200. In this scenario, the standard deviation σ is 1.32, and the standard error $\sigma_{x}$ is 0.22. Throughput remained relatively stable across varying transaction counts. Figure 3b shows that the average delay increased from 0.2 ms to 1.8 ms as the number of nodes grew. Here, the standard deviation σ is 0.26, and the standard error $\sigma_{x}$ is 0.04. Alternative to throughput, average delay remained consistent across different transaction volumes as shown in Figure 3c,d. However, beyond a certain threshold, the throughput plateaued, revealing the system’s scalability limitations.

**Figure 4:**
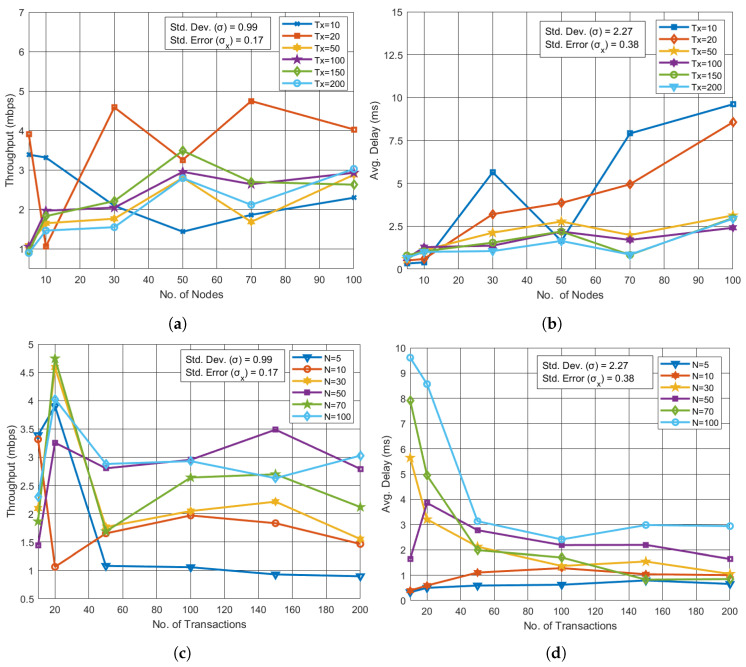
Performance analysis for varying nodes and transactions in UAV network. (**a**)Throughput vs. No. of UAVs with varying Tx. (**b**) Avg. Delay vs. No. of UAVs with varying Tx. (**c**) Throughput vs. No. of Tx with varying UAVs. (**d**) Avg. Delay vs. Tx with varying UAVs.

For the UAV network, throughput showed a positive trend as the number of UAVs and transactions increased. As illustrated in Figure 4a, when the number of UAVs was varied from 5 to 100, throughput experienced a small rise from 1 Mbps to 4 Mbps for fixed transaction counts of 10, 20, 50, 100, 150, and 200. In this scenario, the standard deviation σ was 0.99, with a standard error $\sigma_{x}$ of 0.17, indicating stable throughput across varying transaction counts. Meanwhile, Figure 4b reveals that the average delay significantly increased from 0.2 ms to 9.2 ms as the number of UAVs grew. For this case, the standard deviation σ was 2.27, and the standard error $\sigma_{x}$ was 0.38. Figure 4c,d reflect that there are no noticeable changes in throughput and delay with the varying transaction count.

### 5.4. Performance Measurement Through Area and Height

**Figure 5:**
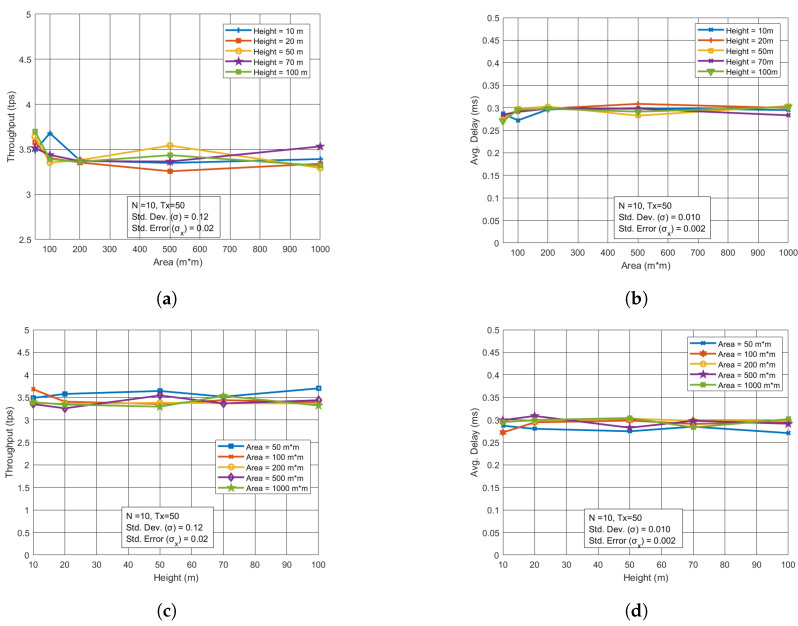
Performance analysis for varying area and height in BC. (**a**) Throughput vs. Area with varying height. (**b**) Avg. Delay vs. Area with varying height. (**c**) Throughput vs. Height with varying area. (**d**) Avg. Delay vs. Height with varying area.

The area and height of the UAV network have a noticeable impact on the performance of the UAV network itself, though their influence on the blockchain network is relatively minimal. We analyzed how changes in area and altitude affect key performance metrics such as throughput and latency. When the area was expanded or UAV altitude varied, these metrics were measured. For the blockchain network, however, changes in area and height had little effect on throughput and latency, as shown in Figure 5a–d. With a fixed number of UAVs (10 in this case) and a fixed number of transactions (50 transactions), variations in area or height did not significantly alter blockchain performance. This suggests that while UAV network performance is more sensitive to these changes, the blockchain network remains largely unaffected.

**Figure 6:**
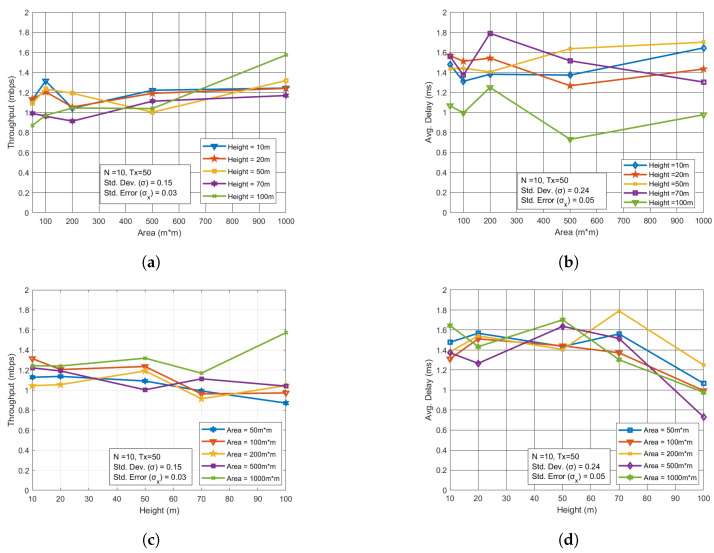
Performance analysis for varying area and height in UAV network. (**a**) Throughput vs. Area with varying height. (**b**) Avg. Delay vs. Area with varying height. (**c**) Throughput vs. Height with varying area. (**d**) Avg. Delay vs. Height with varying area.

We observed that there was no significant increase or decrease in throughput when UAVs operated in larger areas or at higher altitudes. However, the delay did increase with larger areas and higher altitudes due to transmission times, which led to a degradation in the performance of the UAV network. As shown in Figure 6a,c, changes in area and altitude had minimal impact on the network’s throughput. However, Figure 6b,d, illustrate that there was some effect on the delay, indicating that while throughput remained relatively stable, the performance of the UAV network was impacted by the increased delay in larger areas and at higher altitudes.

### 5.5. Performance Measurement Through Data Rate

**Figure 7:**
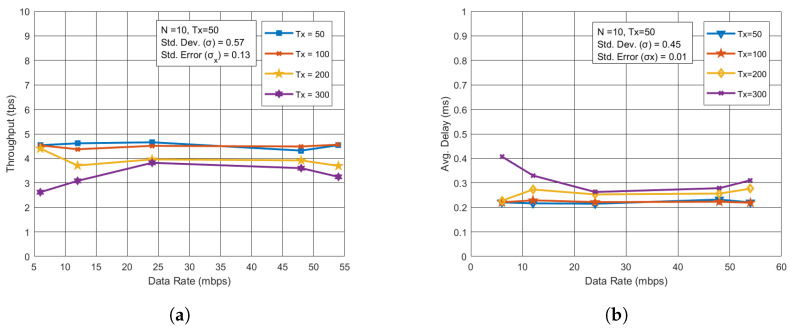
Performance analysis for varying data rates in BC. (**a**) Throughput vs. Data rate with varying Tx. (**b**) Avg. Delay vs. Data rate with varying Tx.

**Figure 8:**
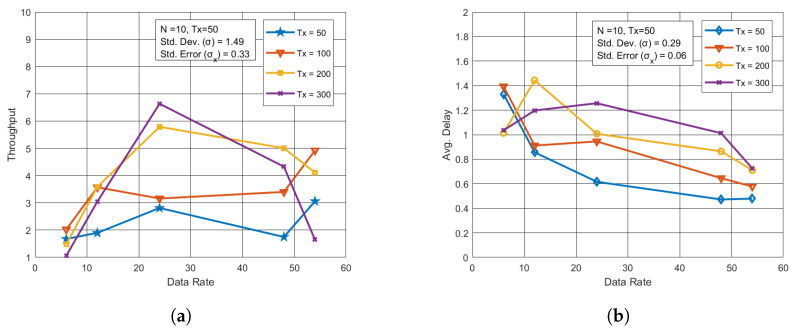
Performance analysis for varying data rates in UAV network. (**a**) Throughput vs. Data rate with varying Tx. (**b**) Avg. Delay vs. Data rates with varying Tx.

The impact of different data rates on throughput and latency was also examined. In the case of the blockchain network, higher data rates had minimal effect on throughput and latency, as depicted in Figure 7a,b. However, for the UAV network, higher data rates led to improved throughput, allowing more data to be transmitted per second, as shown in Figure 8a. Additionally, lower latency was observed at higher data rates in Figure 8b, as transactions were processed more quickly.

## 6. Security Analysis and Verification of the Protocol

**Figure 9:**
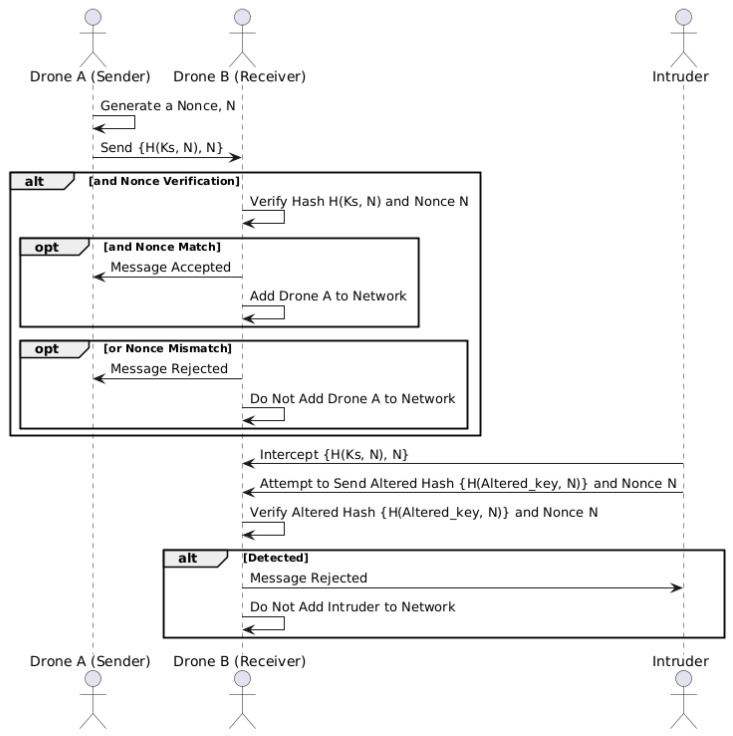
Secure authentication protocol in UAV network.

Given the sensitive nature of UAV communication within a blockchain framework, it is crucial to ensure that the proposed protocol is secure against malicious entities. The protocol must prevent unauthorized access and data manipulation, ensuring that only authenticated UAVs can participate in the network. Figure 9 depicts the sequence diagram for secure authentication in UAV network. To evaluate the security of our protocol, we conducted a formal analysis using the Automated Validation of Internet Security Protocols and Applications (AVISPA) [30] tool. AVISPA is a widely adopted verifier that uses mathematical logic to assess the security properties of protocols.

In AVISPA, protocols are specified in High-Level Protocol Specification Language (HLPSL) [31], allowing for a clear definition of roles and interactions. HLPSL enables the specification of goals such as secrecy and authentication, which are crucial for ensuring data integrity and confidentiality in UAV networks. These interactions are described in a structured manner, following the standard Alice–Bob notation to represent secure communication flows between entities.

The protocol was verified using AVISPA’s OFMC and CL-AtSe [32] backends, both of which confirmed that the protocol is secure under the conditions analyzed:

- OFMC Verification: The OFMC (On-the-Fly Model Checking) backend results indicate that the protocol is **SAFE**, confirming that it meets the defined security goals within a bounded session model. The analysis found no reachable states that violate secrecy or authentication properties, thereby ensuring that the protocol operates securely under normal and adversarial conditions.
- CL-AtSe Verification: Additional verification using the CL-AtSe backend provided further assurance of the protocol’s security. The protocol is marked as **SAFE**, with no unsafe states reachable. This confirms that the protocol maintains its security properties when subjected to various simulated attack scenarios.

### 6.1. Integrity and Authentication

The protocol utilizes a hashing mechanism to ensure both data integrity and authentication. In this setup, ‘role_A’ (the sender) selects a nonce (*N*) and computes the hash of the nonce using a hash function ‘*H*’ and a pre-distributed secret key ($K_{s}$). This hashed message is then transmitted to ‘role_B’ (the receiver). The receiver verifies the integrity of the received message by comparing the transmitted hash and the newly computed hash using the same nonce and secret key.

The use of a hash message in the proposed system serves two critical functions:

1. Integrity: The hash function ensures that any alteration in the hash message (nonce) during transmission results in a different hash, which is detected by ‘role_B’. This mechanism protects against data tampering and guarantees that the transmitted data have not been modified.
2. Authentication: By verifying the hash, ‘role_B’ can confirm that the message originated by ‘role_A’ and has not been manipulated by an intruder. To generate a valid hash, the intruder must have access to the secret key ($K_{s}$) which is impossible without compromising the drone. This mechanism ensures that only authorized UAVs can join and communicate within the network.

### 6.2. Secrecy and Authentication Analysis

To ensure that the protocol maintains confidentiality and authentication, we defined two primary security goals in HLPSL:

1. Secrecy: The protocol includes a secrecy goal to protect sensitive information. The protocol ensures that the secret key ‘$K_{s}$’ remains confidential between the roles ‘A’ and ‘B’. The AVISPA verification confirms that this secrecy property holds, ensuring that no unauthorized party can access the message content.
2. Authentication: Authentication goals were established to confirm that each entity interacting within the protocol is legitimate. In our protocol, the authenticity of each UAV is verified through the hash mechanism, which ensures that only messages from verified senders are accepted. The AVISPA analysis confirms that this authentication goal is satisfied, providing assurance against impersonation attacks.

The protocol was also tested for resilience against intruder attacks and replay attacks. In AVISPA, the intruder is modelled with knowledge of certain values and the ability to intercept messages. However, due to the hashing mechanism, the protocol can perform the following:

- Intruder Resistance: The intruder’s attempts to alter or replay the hashed message are ineffective. The protocol discards any manipulated messages, as a hash mismatch alerts ‘role_A’ to potential tampering. This ensures that intercepted messages cannot be altered or reused without detection.
- Replay Attack [33] Mitigation: The protocol’s resistance to replay attacks is achieved by generating a unique hash for each communication instance. This hash is linked directly to the specific message content, making it impossible for an intruder to replay previous messages successfully. The AVISPA simulation confirmed that the protocol is resilient to replay attempts, as each hash is verified for authenticity and freshness.

**Figure 10:**
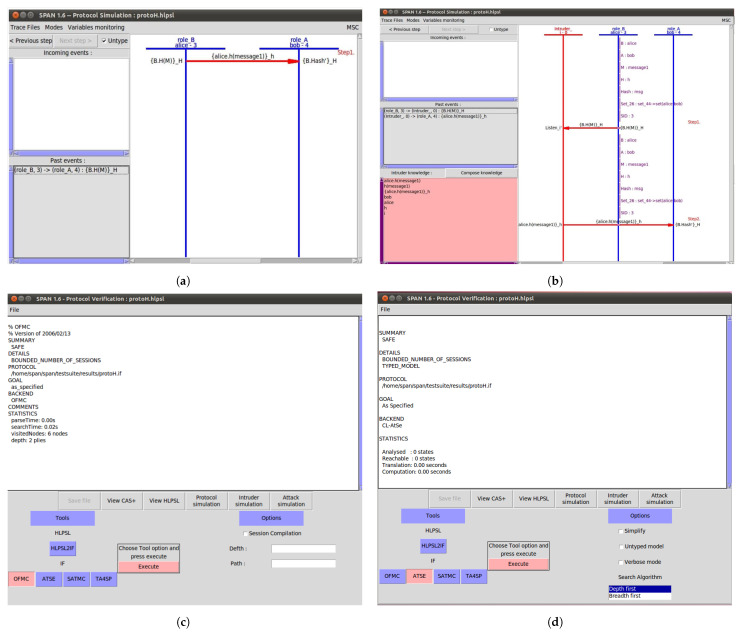
Protocol validation using AVISPA.(**a**) Secrecy verification between A and B. (**b**) Replay attack mitigation using our protocol. (**c**) Protocol verifies as safe for OFMC. (**d**) Protocol verifies as safe for ATSE.

The AVISPA results, shown in Figure 10, demonstrate that the protocol successfully meets its security objectives. The AVISPA analysis validates that our protocol achieves its security goals, ensuring data confidentiality, integrity, and authentication within a UAV blockchain network. The protocol effectively prevents unauthorized access and data manipulation, providing a secure framework for UAV communication.

## 7. Conclusions

This study analyzed the performance of private blockchain integration in UAV networks, focusing on key metrics such as throughput, latency, scalability, and the impact of network parameters like area, altitude, and data rate. Through extensive experiments, we demonstrated that private blockchain offers a feasible solution for decentralized and secure UAV network management, although certain trade-offs exist, particularly in terms of scalability and latency. These findings highlight the potential and limitations of integrating blockchain technology into UAV networks, with scalability and latency being key challenges that need to be addressed in future research. As the proposed system implements a simple one-way authentication mechanism, future work will focus on integrating a robust two-way authentication mechanism into the proposed architecture. In our future research, we also aim to integrate machine learning (ML) and artificial intelligence (AI) with blockchain to enhance the efficiency and trust in UAV networks. This integration has the potential to enable intelligent data analysis, real-time threat detection, and secure autonomous operations. We also aim to compare the performance of blockchain-based UAV networks with traditional, centralized UAV networks to understand their relative efficiency, scalability, security, and potential impact on operational effectiveness.

## Figures and Tables

**Table 1:** Simulation Parameters and their Values.

Simulation Parameters	Values
Simulator Used	NS-3.36.1
Channel Type	Wireless (YANS Wi-Fi Channel)
Radio Range	Configured using RandomBoxPositionAllocator
MAC Protocol	MAC/802.11ac
Mobility Model	Gauss-Markov Mobility Model
Node Speed	Mean Velocity: 800 to 1200 m/s
Number of Zones	Not explicitly defined
Number of Nodes	1 AP + Configurable number of STAs (e.g., 100)
Number of Drones/Controllers	Not explicitly defined
Traffic Type	UDP Echo (Client-Server)
Simulation Time	100 s
Area	100 m × 100 m × 100 m
Packet Size	512 Bytes
Block Size	0.1 MB to 1 MB (See the blockchain log size)
Block Header Size	80 Bytes
Number of Miners	Variable (depends on node configuration)
Data Rate	OFDM Rates (6, 9, 12, 18, 24, 36, 48, 54 Mbps)
Flow Monitoring	Enabled (FlowMonitor)
Network Animation	Enabled (NetAnim)

## Data Availability

The data supporting the findings of this study are available from the corresponding author upon reasonable request.
